# Elliptical Subject Specific model for respiratory motion

**DOI:** 10.1186/1532-429X-14-S1-P267

**Published:** 2012-02-01

**Authors:** Ian Burger, Ernesta M Meintjes, Jennifer Keegan, David N Firmin

**Affiliations:** 1 MRC/UCT Medical Imaging Research Unit, Department of Human Biology, University of Cape Town, Cape Town, South Africa; 2 CMR Unit, Royal Brompton Hospital Trust, London, UK; 3 Imperial College London, London, UK

## Summary

Novel non-rigid Elliptical model for Subject Specific motion correction for respiratory motion.

## Background

Respiratory motion of the heart poses a problem for high resolution cardiac MR imaging. Prospective slice following uses the navigator position immediately prior to the imaging segment to correct the slice positions throughout the segment [1]. The navigator is typically placed over the right hemi-diaphragm and a fixed correction factor is used to adjust for the difference to the motion of the heart. The relationship between the motion of the heart and the superior-inferior motion of the diaphragm is approximately linear although highly subject specific, with an element of hysteresis [2]. We investigated a more complex model to incorporate non rigid transformation of the heart as well as hysteresis.

## Methods

In this study we investigated the possibility of modelling the respiratory motion of the heart non-rigidly including the hysteretic effect. Nine healthy subjects received MR scanning according to protocols that had been approved by the IRB’s of Imperial College London and University of Cape Town. A single-shot 2D image of the heart, preceded by a navigator, was acquired during each cardiac cycle for ninety cycles. Coronal and sagittal images were acquired with in-plane resolution 1.61x1.88 mm after interpolation.

Images were cropped, segmented and registered non-rigidly to the first image in the series. The first navigator value was used as the reference. In order to account for the hysteresis, respiratory motion was modelled using an ellipse superimposed on a straight line. A cost function set to minimize the RMS-error of the model was used to calibrate each component of the affine matrix model. The model was tested by comparing transformation matrices computed using the navigator outputs for each cardiac cycle and our subject-specific respiratory model to transformation matrices obtained using co-registration.

## Results

**Figure 1:**
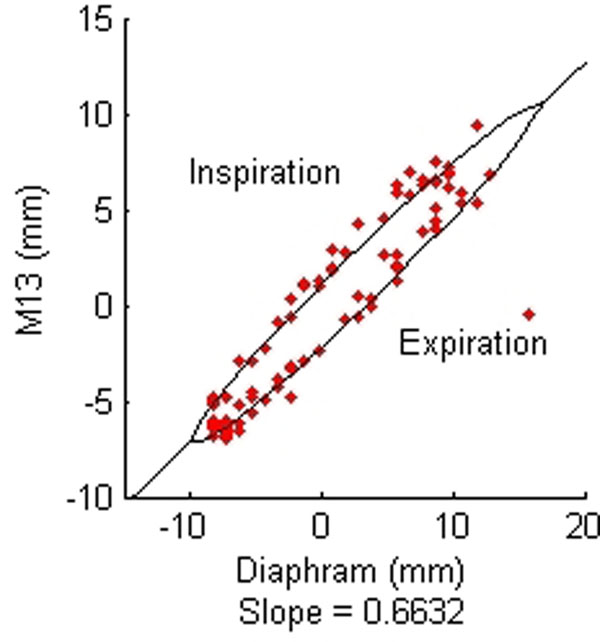
One of the components of the affine transformation matrix plotted against the navigator values for one of the subjects (Coronal)

**Figure 2:**
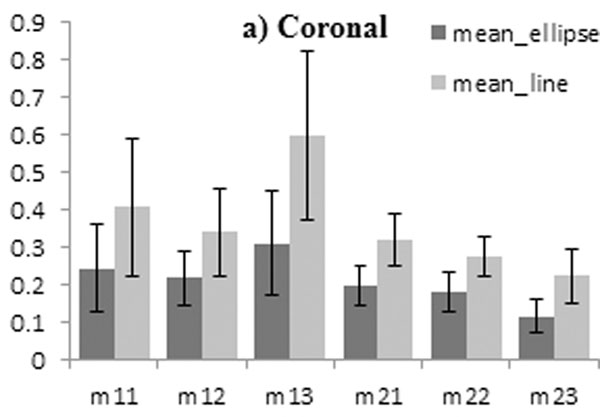
The mean RMS error ± standard deviation of the components of the matrix for straight line and elliptical model in the coronal plane

Figure 1 shows one of the components plotted against the navigator values for one of the subjects and the curve fitted. Figure 2 shows the mean error ± standard deviation of the components of the matrix for straight line and elliptical models in the coronal plane.

## Conclusions

The process was completely automated in order to be replicable online to allow rapid construction of a subject-specific model from a short pre-scan. The results show that the elliptical model performs better than the linear affine model.

## Funding

The South African Research Chairs Initiative of the Department of Science and Technology and National Research Foundation of South Africa.

Medical Research Council of South Africa, South Africa / Norway Research Collaboration Programme, and the University of Cape Town.

